# Arabic validation of the “Mental Health Knowledge Schedule” and the “Reported and Intended Behavior Scale”

**DOI:** 10.3389/fpsyt.2023.1241611

**Published:** 2023-10-19

**Authors:** Maryem Ben Amor, Yosra Zgueb, Emna Bouguira, Amani Metsahel, Amina Aissa, Graham Thonicroft, Uta Ouali

**Affiliations:** ^1^Department of Psychiatry A, Razi University Hospital, La Manouba, Tunisia; ^2^Faculty of Medicine of Tunis, University of Tunis El Manar, Tunis, Tunisia; ^3^Research Laboratory LR18SP03, Manouba, Tunisia; ^4^Pôle G01 Etablissement Publique de santé Alsace Nord, Strasbourg, France; ^5^Centre for Global Mental Health and Centre for Implementation Science, Health Service and Population Research Department, Institute of Psychiatry, Psychology and Neuroscience, King’s College London, London, United Kingdom

**Keywords:** stigma, mental illness, validation, students, surveys and questionnaires

## Abstract

**Objectives:**

Mental illness affects one in eight people in the world according to the WHO. It is a leading cause of morbidity and a major public health problem. Stigma harms the quality of life of people with mental illness. This study aimed at validating the Arabic version of the Mental Health Knowledge Schedule (MAKS) and the Reported and Intended Behavior Scale (RIBS) in a sample of Tunisian students and determining socio-demographic and clinical factors correlated with stigma.

**Methods:**

This cross-sectional study was conducted on 2,501 Tunisian students who filled in the MAKS, the RIBS, and a sociodemographic and clinical questionnaire. The validation of the questionnaires in Arabic was carried out using the validity criteria: face and content validity, reliability, and construct validity. Next, the associations between stigma and sample characteristics have been studied using multivariate linear regression.

**Results:**

Face and content validity of the measures MAKS and RIBS were satisfactory, with adequate internal consistency. There were significant positive correlations between the items and scales, and test–retest reliability was excellent. The internal validity showed that the items were well-aligned with the intended factors, and the external validity revealed a significant positive relationship between the MAKS and RIBS. Besides, gender, the field of study, psychiatric history, and contact with someone with a mental illness were all contributing factors to mental illness stigma. Additionally, men performed better than women in terms of behavior toward people with mental illness, while women had a greater level of knowledge about mental health.

**Conclusion:**

The Arabic versions of the MAKS and RIBS have appropriate psychometric properties, making them effective tools for evaluating mental illness stigma. With multiple factors contributing to this issue, these instruments can help focus anti-stigma efforts and promote a more inclusive society.

## 1. Introduction

Stigma against mental illness is a universal issue considered to be the main barrier to access mental health care (1). It significantly harms the quality of life of people with mental illness and its negative effects can be seen in various areas of their life, including difficulty finding and maintaining housing and employment, limited social connections, and finances (2).

Stigmatization can arise because of several attributes of a person, namely differences in visible physical manifestations (deafness, blindness), origins (ethnicity, religion, race…), or behaviors (3). However, compared to these, mental illnesses are often more stigmatized, which has been called the ultimate stigma (4).

Worldwide, the burden of mental illness continues to grow significantly with significant health impacts and major socioeconomic and human rights consequences according to WHO in 2019. Indeed, mental illness nowadays affects about 970 million people around the globe (5).

To better understand stigma, it is important to consider its three constructs: knowledge (ignorance), attitudes (prejudice), and behavior (discrimination) (6, 7). Surprisingly, when it comes to knowledge, the public understanding of the biological underpinnings of mental illness does not seem to lead to greater social acceptance of these individuals. Indeed, these individuals are still described as “dangerous” and “unpredictable,” which increases social distance (8).

Several studies have shown that mental health knowledge specific to symptom recognition, treatment efficacy, and help-seeking can facilitate understanding when communicating with clinicians and decrease fear and embarrassment when interacting with people with mental illness (9, 10). Thus, it can play a key role in influencing behaviors and attitudes (11).

Rates of anticipated and experienced discrimination among people with mental illness are consistently high (12). Not only do they have to deal with the handicap inflicted by the symptoms of the disease, but they must face the harsh judgments made by society in their daily lives (13). In a cross-sectional study comparing public beliefs and attitudes toward schizophrenia in Central Europe (Germany) and North Africa (Tunisia), individuals with schizophrenia in Tunisia were found to be more accepted in distant relationships, such as being a neighbor or colleague, but faced stronger rejection in culturally significant family roles, such as marrying into the family or taking care of children, risking exclusion (14).

Enhancing the public’s understanding of mental health can have a positive effect on reducing stigma and social exclusion, increasing willingness to seek help, and ultimately improving individuals’ adherence to treatment in the future (15).

Although the number of studies assessing stigma and testing interventions to reduce stigma is continually growing in the Arab world, their number is still low compared to Western countries. In addition, there is a lack of contextually adapted and validated instruments to measure stigma and assess the efficacy of anti-stigma interventions in the Arab world.

The Mental Health Knowledge Schedule (MAKS) and the Reported and Intended Behavior Scale (RIBS) were adapted to Arabic in the context of the “INDIGO” partnership research program whose aim is to increase the understanding of mechanisms behind stigma and to develop interventions to reduce stigma toward individuals with mental health issues in low-and middle-income countries (LMICs) (16).

Unlike previous measures that focused on evaluating knowledge and behavior within specific populations or for specific diagnoses, the MAKS and RIBS were developed as part of the UK Time To Change (TTC) anti-stigma campaign 2008–2012 to evaluate the contribution of interventions to knowledge and behavior change of the general public and to allow the comparability of results (17). This study aimed at validating the Arabic versions of the MAKS and the RIBS in a sample of Tunisian students and identifying socio-demographic and clinical factors associated with stereotypes and stigmatizing behavior in this student sample.

## 2. Materials and methods

### 2.1. Sample

We conducted a descriptive and validation study on 2,501 Tunisian students enrolled in public and private universities in different regions of Tunisia during the academic year 2020–2021. Our study did not include foreign students, medical students, and participants who had completed their university studies.

### 2.2. Methods of recruitment

We asked the student delegates of each institution to distribute the form via the mailing lists of students of each institution. Thus, we were able to target students from different fields of study (Arts, Economics and Management, Literature, and Sciences) enrolled in both public and private sector institutions in all regions of Tunisia between July and November 2021. The form was accompanied by a description containing information on the purpose and content of the study, as well as on the confidentiality of the data and its use for purely scientific purposes. Accessing the form and answering the questionnaire indicated the consent of the candidate. Each student could only access the questionnaire once to avoid data redundancy. If a student answers “No” to the question “Are you a student?,” he/she will be automatically directed to the end of the questionnaire. Candidates could not send their answers if they did not complete the questionnaire. We have also invited 110 subjects to participate in the test–retest study after a random selection. Their responses were combined with their initial results to compare them. The time to answer the form varied between 5 and 10 min. All results were automatically recorded in an excel file accessible only to the author of the form.

### 2.3. Measurement

#### 2.3.1. MAKS

This questionnaire was designed by S. Evans-Lacko and published in 2010 (15). It contains two sub-scales each consisting of six items scored on a Likert scale. The first six items refer to mental health knowledge. Items 7–12 assess whether participants qualify the following conditions: depression, stress, schizophrenia, bipolar disorder, drug addiction, or grief as mental illness (15). It has already been validated in French, Italian, Persian, and Kiswahili languages (18–21).

#### 2.3.2. RIBS

The RIBS was developed to enhance the assessment of anti-stigma interventions by encouraging the integration of behavioral outcomes (22) and has been validated in French, Italian, Japanese, Chinese, and Brazilian (18, 23–26). It consists of two sub-scales exploring four different areas: living with, working with, living near, and pursuing a relationship with someone with a mental health problem. It contains eight items, the first four of which explore the prevalence of reported or actual behavior with three possible responses “No,” “Yes,” and “I do not know,” and the second sub-scale with four items evaluates future intentions in the four areas described above and are scored on a Likert scale (22). This distribution enables us to understand how reported behavior may influence intended behavior.

#### 2.3.3. Items coding

All items which were assessed using the Likert Scale were coded from 1: “Strongly disagree” to 5: “Strongly agree.” “Do not know” was coded as neutral (i.e., 3). Items 6, 8, and 12 of the MAKS were reverse-coded. No score value was assigned to items 1–4 of RIBS because they only calculate the prevalence of behaviors. The total score was calculated by summing the values of the responses. A higher score of responses to MAKS and/or RIBS reflects a better understanding of mental illness and a higher willingness to engage in positive behaviors toward people with mental illness (15, 22).

### 2.4. Procedure

#### 2.4.1. Data analysis

Data analysis was performed using SPSS version 26.0 statistical software after importing the data from the Excel document that was recorded from the form responses.

We proceeded to a descriptive study of the population according to the different criteria: quantitative variables were studied using means (M) or medians, and qualitative variables were described using percentages (%) and standard deviations (SD). Determinants of mental illness stigma were analyzed using multivariate linear regression. In all statistical tests, the threshold of statistical significance was “*p* < 0.05.”

#### 2.4.2. Translation

We translated the MAKS and the RIBS from the original version to easily understandable standard Arabic based on the “back-translation “method. The first step was the translation from English to standard Arabic. Then, this version was evaluated by a committee of experts composed of psychiatrists, psychologists, and psychiatric nurses from Razi Hospital as well as of service users. Second, we realized back-translation from Arabic to English. Finally, the original and back-translated versions were subjected to a comparative analysis. The purpose of this step was to verify the adequacy of the translation and the adaptation of the items to the socio-cultural context. The preliminary version obtained at the end of this stage was studied by the committee of experts who evaluated the clarity, discrimination, and relevance of the MAKS and RIBS items. The resulting version was pre-tested with a sample of 30 individuals from the target population.

#### 2.4.3. Validity study

##### 2.4.3.1. Reliability

We analyzed the reliability of the two scales using “Cronbach’s alpha” coefficient, whose value can vary between 0 and 1. Internal consistency is considered satisfactory starting from 0.7; however, above 0.9, it could indicate a certain redundancy of the items (27). To strengthen our study, we studied the inter-item and total inter-item correlation using the Pearson coefficient. This step assesses the strength of the link between the items within the same scale (27).

We also studied the test–retest reliability using the “intra-class coefficient” (ICC) and the “paired samples” to verify the consistency of the results over a 1 month interval.

##### 2.4.3.2. Construct validity

To establish internal construct validity, we opted for a confirmatory factor analysis (CFA): we set a number of factors according to the number of dimensions we wanted to identify, and the proposed model was retained or not according to the “fit measures” obtained (28). Then, we associated a step studying the factorial solutions of each questionnaire. This step was carried out using two tools: the “Kayser-Meyer-Olkin” (KMO) measure which should exceed 0.6 for factorability (29) and the “Barlett sphericity test” which requires at least five individuals per variable given its high sensitivity to the number of individuals (30).

To test convergent validity and as, at the time of the study, no validated scales in Arabic assessing the stigma of mental illness in the general population existed, we studied the correlation between the MAKS and the RIBS using the Pearson coefficient after having validated each of the two scales.

## 3. Results

### 3.1. Sample characteristics

We included 2,501 participants. Their sociodemographic and clinical characteristics are presented in Table 1.

**Table 1:** General characteristics of the participants included in the study (*N* = 2,501).

Participant’s characteristic	M	SD
Mean age	21.57	2.55
Sex-ratio male/female	0.37	
	N	%
Family psychiatric history	428	17
Personal psychiatric history	440	17.6
Tobacco use	522	20.9
Considers him/herself as religious	1,892	75.6
Field of study		
Science and technology	1,468	58.7
Literature	436	17.4
Economics and management	381	15.2
Arts	120	4.8

### 3.2. Distribution of participants’ responses

The median MAKS score was 45 out of 60 with a range of 30–56. The distribution of participants’ responses to the MAKS is detailed in Table 2.

**Table 2:** Distribution of participants’ responses to the MAKS.

MAKS	Strongly disagree	Disagree	Neither agree nor disagree/ I do not know	Agree	Strongly agree
Most people with mental health problems want to have paid employment.	5.30%	10.40%	60.20%	15.40%	8.70%
If a friend had a mental health problem, I know what advice to give them to get professional help.	1.20%	3.10%	12%	31.20%	52.50%
Medication can be an effective treatment for people with mental health problems.	7.70%	16.90%	18.30%	38.50%	18.60%
Psychotherapy (e.g., counseling or talking therapy) can be an effective treatment for people with mental health problems.	0.90%	3.20%	5.90%	25.30%	64.70%
People with severe mental health problems can fully recover.	4%	8.90%	18.40%	35.30%	33.50%
Most people with mental health problems go to a healthcare professional to get help.	17.40%	21.60%	29.70%	21.70%	9.70%
Depression	1.40%	4.20%	3.60%	17.20%	73.60%
Stress	5.10%	11.30%	12.50%	30.80%	40.30%
Schizophrenia	0.60%	1.50%	3.50%	8.80%	85.60%
Bipolar disorder (maniac depression)	0.70%	3.30%	7.50%	13.50%	75.10%
Drug addiction	6.60%	11.10%	19.40%	24.80%	38.20%
Grief	10.20%	15.90%	21%	27.20%	25.70%

The first four items of the RIBS are not part of the behavioral assessment. However, they provide information about the prevalence of behaviors in each of the four contexts. Thus, the median RIBS score was calculated using the scores of items 5–8, and it was equal to 15 out of 20 ranging from 4–20. The distribution of participants’ responses to the RIBS is illustrated in Table 3.

**Table 3:** Distribution of participants’ responses to the RIBS.

RIBS (Reported behavior)	Yes	No/ I do not know			
Are you currently living with, or have you ever lived with, someone with a mental health problem?	40%	60%			
Are you currently working with, or have you ever worked with, someone with a mental health problem?	24%	76%			
Do you currently have, or have you ever had, a neighbor with a mental health problem?	34%	66%			
Do you currently have, or have you ever had, a close friend with a mental health problem?	51%	49%			
RIBS (Intended behavior)	Strongly disagree	Disagree	Neither agree nor disagree/ I do not know	Agree	Strongly agree
In the future, I would be willing to live with someone with a mental health problem.	14%	11.80%	24.50%	30.10%	19.60%
In the future, I would be willing to work with someone with a mental health problem.	13.20%	11.90%	21.50%	30.90%	22.50%
In the future, I would be willing to live nearby to someone with a mental health problem.	10.60%	9.80%	21.70%	31.60%	26.40%
In the future, I would be willing to continue a relationship with a friend who developed a mental health problem.	3.10%	3.30%	10%	23%	60.70%

### 3.3. Validity study

#### 3.3.1. Content validity

Among the 12 items of the MAKS scale, 4 items were discussed by experts: items 9 (“Schizophrenia”), 10 (“Bipolar disorder”), and 12 (“Grief”) were reworded to make them more understandable and item 4 (“Psychotherapy (e.g., counseling or talking therapy) can be an effective treatment for people with mental health problems.”) was reworded with a version more adapted to the Arabic and Tunisian context. For RIBS, the title was reworded with terms better understood by the local context. At the pre-test stage, only item 1 (“Most people with mental health problems want to have paid employment.”) of the MAKS was ambiguous for one participant, and we remedied this problem by adding an explanation to the questionnaire statement (“Most people with mental health problems want to have paid employment, just as anybody”).

#### 3.3.2. Reliability

The internal consistency of the two questionnaires was evaluated by “Cronbach’s alpha,” which was 0.56 for MAKS and 0.83 for RIBS. The “inter-item,” “item-total” and “inter-dimensional correlation” studies showed a significantly positive correlation between the different items of each scale and the totals of each sub-scale, indicating good overall reliability. Regarding test–retest reliability, the intra-class coefficient was 0.882 and 0.996 for the MAKS and RIBS total scores, respectively, indicating excellent concordance and thus good stability of responses over time. This result was supported by the paired samples *T*-test for each of the two questionnaires (Table 4).

**Table 4:** Validity tests of both MAKS and RIBS.

Scale	Internal consistency	Inter-dimension correlation	Test–retest reliability		Internal validity		Convergent validity
	Cronbach’s alpha	Pearson’s coefficient	Intra-class correlation coefficient	Paired samples *T*-tests (*p*)	KMO index	Percentage of total variance explained	Pearson’s correlation between MAKS and RIBS
MAKS	0.56	0.170^*^	0.882	0.456	0.632	31.58%	0.154^*^
RIBS	0.83	0.112^*^	0.996	0.059	0.763	52.28%	

*The correlation is significant at the 0.05 level (two-tailed).

#### 3.3.3. Construct validity

The KMO index was 0.632 for the MAKS and 0.763 for the RIBS. The total variance explained by the two factors was 31.58% for the MAKS and 52.28% for the RIBS. All items saturated in the expected factor of each scale as for the original version. Thus, the same distribution was maintained for both the MAKS and the RIBS. Regarding the convergent validity study, Pearson’s coefficient was 0.154 between the scores of the RIBS and the MAKS (Table 4).

### 3.4. Determinants of mental health stigma

Multivariate linear regression analysis showed that gender, psychiatric history, contact with a person with mental illness, and the artistic field of study were found to be independently associated with mental health knowledge and intended behavior toward people with mental illness (Table 5).

**Table 5:** Multivariate regression analysis of the determinants of mental health stigma.

Determinants	MAKS Score as a dependent variable		RIBS Score as a dependent variable	
	β	*p*	β	*p*
(Constant)	44.623	0.000	13.783	0.000
Male gender	−0.635	0.000	0.409	0.033
Personal psychiatric history	0.273	0.158	1.677	0.000
Family psychiatric history	0.297	0.140	0.478	0.034
Artistic field of study	−0.748	0.044	−0.201	0.628
Psychoactive substance use	0.158	0.386	0.372	0.068
Considers him/herself as religious	−0.036	0.858	−0.292	0.198
Contact with a person with a mental illness	0.450	0.005	0.736	0.000

## 4. Discussion

### 4.1. Validity study

The MAKS and RIBS have recently been validated in multiple languages (18–21, 23–26). However, no measurement tool assessing knowledge and behavior toward mental illness in the general population has been validated in Arabic, except for a questionnaire assessing attitudes toward patients with schizophrenia entitled “Attribution Questionnaire” which has been validated in Arabic in a population of Tunisian university students (31). Our validation study will add two valuable tools to assess mental health related knowledge and behavior in Arabic-speaking general population samples. In addition, the MAKS and RIBS differ from the previously validated measurement tool in that they address mental health problems in general, without specifying a diagnosis.

In our study, Cronbach’s alpha was equal to 0.56. While certain authors argue that a reliability score of at least 0.7 is necessary (32, 33), the threshold of 0.56 remains acceptable based on the standards established by George and Mallery (34), especially since the MAKS is not considered a scale, and each of its items evaluates knowledge on a specific domain. This has already been discussed in previous versions. The MAKS was therefore considered more as an indicator of trends in responses (15). Thus, the alpha coefficient in the original version was 0.65. Indeed, it is important to keep in mind that the results of the various statistical tests may be less strong than those of the original version due to the linguistic and cultural differences between the two versions. Moreover, the inter-item correlation coefficients of the MAKS were low, which was also explained by the heterogeneity of the set of items (15).

Regarding the RIBS, our translated Arabic version had an internal consistency very similar to that of the original version (0.85) (22). The inter-item and inter-dimension correlation studies showed a significantly positive correlation between the different items of the MAKS and the RIBS and between the two subscales of each scale.

The ICC of the MAKS and RIBS total score indicated excellent concordance and thus good stability of responses over time.

Pearson’s correlation between the MAKS and the RIBS was comparable to the one of the French and Kiswahili versions (18, 21).

### 4.2. Mental health stigma in Tunisian students

Our sample size was 2,501, largely exceeding that of the original validation study which included a total of 403 students (15, 22) and that of other validation studies (18–21, 23–26), with a good representativeness of the sample. However, as our study targeted only adolescents and young adults, it would be useful to study “knowledge” and “behavior” using MAKS and RIBS in all age groups. The online questionnaire allowed us to maximize the number of participants in a shorter time, target both public and private institutions from different regions of Tunisia, ensure anonymity, and limit social desirability bias.

The responses obtained for each of the items of the MAKS and the RIBS were close to those of the original version, except for item 1 of the MAKS where 60.2% answered “do not know” or “neither agree nor disagree.” In Tunisian society, there could be a lack of understanding or education about mental illness and its effects on individuals’ ability to work. Indeed, this item was removed from the French validation study of the MAKS (18). However, we decided to keep it in our study as we believe it is relevant in the Tunisian social context given that work is considered as a means of upward social mobility. Therefore, we should think about studies and discussion forums that are relevant to employment among people with mental illness.

Women tended to have significantly higher scores on mental health knowledge (β = 0.635; *p* < 0.001), such as employment among people with mental illness, the effectiveness of treatments, help-seeking, and the classification of various mental health conditions compared to men. On the other hand, men tended to have higher scores on intended behavior (β = 0.409; *p* = 0.033), which suggests that they are less discriminating than women and may engage in more positive interactions with individuals with mental illness. This relation has not been clearly addressed in the literature. Nevertheless, a significant association between attitudes toward mental illness and the female gender has been explained by the fact that women are more empathetic, open-minded, and positive thus showing less stigma (35, 36), but may also be more fearful and avoidant, of people with mental illness, than men (37).

Besides, participants who studied “Art” had lower scores on mental health knowledge (β = −0.748; *p* = 0.044). Indeed, the scientific and literary fields in Tunisian institutions offer training that provides a minimum of knowledge about mental health, unlike the “Art” disciplines, which leads us to think about the importance of integrating educational content related to mental health.

In addition, the results show less discrimination toward the mentally ill among participants with a personal (β = 1.67; *p* < 0.001) or family (β = 0.478; *p* = 0.034) psychiatric history. Participants who have been in contact with someone with a mental illness had also higher scores on both mental health knowledge (β = 0.450; *p* = 0.005) and behavior (β = 0.736; *p* < 0.001) toward people with mental illness. This result is consistent with several prior studies which suggested that contact with individuals with mental illness can help reduce stigma (38–41).

These findings emphasize the advantages of having personal interactions and experiences with individuals who have a mental illness, as well as the importance of providing information about mental illness. These factors had a positive impact on the participants’ knowledge and behaviors toward people with mental illness. It is worth considering the statement of Professor Sartorius that successful campaigns can be implemented in any country or region, regardless of its size, economic status, or level of development (42).

### 4.3. Strengths of the study

Significant merits of our study encompass a large sample size of 2,501 participants, outstripping the original version (15, 22) and other validation studies (18–21, 23–26), representing diverse fields of study and institutions across Tunisia. We excluded medical students to limit knowledge bias. We conducted the study online to reach our technologically proficient target population, ensuring a fast response rate, wide regional coverage, anonymity, and reduced social desirability bias.

Additionally, our study suggests valuable tools for assessing mental health knowledge and behavior in all Arabic-speaking general population samples.

Our research would be of great interest in advancing the ongoing assessment of stigma, providing a solid foundation for the development of anti-stigma strategies. Notably, the MAKS and RIBS differ from previously studied measurement tools in that they target the general public and include a broader range of mental health problems, unlike existing tools that focus on specific diagnoses (31).

This greatly enhances the significance of our research for reducing stigma and developing effective strategies.

### 4.4. Limits of the study

The study is cross-sectional, which does not provide information on possible changes over time and on the influence of certain factors on the responses of the same individual.

Our study might have an inherent selection bias, given that it clearly indicated the subject of mental health at the beginning of the online questionnaire. Therefore, it could have attracted a particular profile of individuals that are less stigmatizing or discriminating against mental illness and pay more interest to stigma, hence the relatively high rate of people with personal psychiatric history (17.6%). Furthermore, an online questionnaire does not allow us to estimate the proportion of refusals compared to those who agreed to participate in our study. Thus, the overall scores of mental health knowledge and intended behaviors may be overestimated. Moreover, declarative bias may contribute to the overestimation of the scores.

In addition, our study population is limited to students and findings can therefore not be extrapolated to all of Tunisia’s population. Although an online questionnaire limits social desirability, students may tend to answer Likert scale questions in the same direction to obtain a higher score due to the competitive nature of the student population.

On another note, the MAKS intentionally incorporates items with a multidimensional structure to assess various types of mental health knowledge. As a result, internal coherence and inter-item and item-total correlation are low. This issue has also been discussed in validation studies of previous versions (15).

Finally, in the absence of validated measurement instruments in Arabic that assess mental health stigma in the general population, we assessed external validity by correlating the MAKS and RIBS scales, a methodology previously used in the French, Italian, and Kiswahili versions (18, 19, 21).

## 5. Conclusion

The MAKS and RIBS validated in standard Arabic show good psychometric properties and could therefore be used in all Arabic-speaking populations to compare results and to study common determinants of mental illness stigma.

Mental illness stigma is influenced by multiple factors, including gender, field of study, psychiatric history, and contact with someone with a mental illness.

This study should be extended to the general population with more representative groups by carrying out on-site studies in public spaces or door-to-door to ensure representative samples of the Tunisian population, i.e., wider age groups and more varied intellectual and socio-professional levels.

Further research on mental illness attitudes is necessary to explore potential correlations. Using objective measures can help to track changes in knowledge, attitudes, and behavior over time, which would strengthen and guide efforts to decrease stigma.

## Data availability statement

The raw data supporting the conclusions of this article will be made available by the authors, without undue reservation.

## Ethics statement

The studies involving human participants were reviewed and approved by Ethics Committee of Razi Hospital. Accessing the form and answering the questionnaire indicated the consent of the candidate.

## Author contributions

MB and YZ designed the study. YZ, AA, EB, AM, and UO evaluated the Arab versions of the MAKS and RIBS. Data were collected and interpreted by MB, YZ, and UO who have drafted the work, which was reviewed by all authors. All authors contributed to the article and approved the submitted version.
