# Supplementary material for: Arabic validation of the “Mental Health Knowledge Schedule” and the “Reported and Intended Behavior Scale”
Source: Front Psychiatry. 2023 Oct 19;14:1241611. doi: 10.3389/fpsyt.2023.1241611 (PMC10620497; doi:10.3389/fpsyt.2023.1241611)
Supplement: Supplementary file 1 [file Data_Sheet_1.PDF]

Supplementary Material

Arabic validation of the “Mental Health Knowledge Schedule” and the “Reported and Intended Behaviour Scale”

Maryem Ben Amor[1,2], Yosra Zgueb[1,2,3], Emna Bouguira[4], Amani Metsahel[1], Amina Aissa[1,2,3], Graham Thonicroft[5], Uta Ouali[1,2,3].

1. Department of Psychiatry A, Razi University Hospital, La Manouba, Tunisia.
2. Faculty of Medicine of Tunis, University of Tunis El Manar, Tunis, Tunisia.
3. Research Laboratory LR18SP03
4. Pôle G01 Etablissement Publique de santé Alsace Nord, Brumath, Strasbourg
5. Centre for Global Mental Health and Centre for Implementation Science, Health Service and Population Research Department, Institute of Psychiatry, Psychology and Neuroscience, King’s College London, London, UK

*** Correspondence:** Maryem Ben Amor: benamor.maryem15@gmail.com

1 Supplementary Tables

Supplementary Table 1. Arabic Translation of MAKS' Items

English version of the MAKS	Arabic version of the MAKS
**Mental Health Knowledge Schedule**	**مقياس المعرفة في الصحة النفسية "مكس"**
1. Most people with mental health problems want to have paid employment.	1. إن معظم الناس الذين لديهم مشاكل في الصحة النفسية يحبذون العمل بأجر.
2. If a friend had a mental health problem, I know what advice to give them to get professional help.	2. إذا كان لي صديق يعاني من مشكلة في الصحة النفسية، فأنا أعرفـكيف انصحه ليحصل على المساعدة من ذوي الاختصاص.
3. Medication can be an effective treatment for people with mental health problems.	3. يمكن أن تكون الأدوية علاجا فعالا ًللأشخاص الذين لديهم مشاكل في الصحة النفسية.
4. Psychotherapy (eg counseling or talking therapy) can be an effective treatment for people with mental health problems.	4. العلاج النفسي(بالكلام) يمكن أن يكون علاجاً فعالاللأشخاص الذين لديهم مشاكل في الصحة النفسية.
5. People with severe mental health problems can fully recover.	5. يمكن للأفراد الذين لديهم مشاكل نفسية صعبة أن يتعافوا تماما.
6. Most people with mental health problems go to a healthcare professional to get help.	6. إن معظم الأشخاص الذين لديهم مشاكل في الصحة النفسية يذهبون إلى أخصائيي الرعاية الصحية.
7. Depression	7. الإكتئاب
8. Stress	8. الضغط النفسي \ التوتر
9. Schizophrenia	9. الفصام الذهاني\ (شزوفرينيا)
10. Bipolar disorder (maniac depression)	10. الإضطراب الثنائي القطبي \الإضطراب في المزاج
11. Drug addiction	11. الإدمان على المخدرات
12. Grief	12. الحداد (في حال فقدان شخص)

Supplementary Table 2. Arabic translation of RIBS' items

English version of the RIBS	Arabic version of the RIBS
**Reported and Intended Behaviour Scale**	**مقياس السلوكيات المعلنة و النوايا « ربس»**
1. Are you currently living with, or have you ever lived with, someone with a mental health problem?	1. هل عشت سابقا أوتعيش الآن مع شخص لديه مشاكل في الصحة النفسية؟
2. Are you currently working with, or have you ever worked with, someone with a mental health problem?	2. هل عملت سابقاأو تعمل الآن مع شخص لديه مشاكل في الصحة النفسية؟
3. Do you currently have, or have you ever had, a neighbor with a mental health problem?	3. هل كان لديك سابقا أو الآن جار لديه مشاكل في الصحة النفسية ؟
4. Do you currently have, or have you ever had, a close friend with a mental health problem?	4. هل كان لديك سابقا أو الآنصديقمقرب لديه مشاكل فيالصحةالنفسية ؟
5. In the future, I would be willing to live with someone with a mental health problem.	5. فيالمستقبل، أنا على استعداد للعيشمع شخص لديه مشاكل في الصحة النفسية (العائلة,قرين, زوج, قريب)
6. In the future, I would be willing to work with someone with a mental health problem.	6. في المستقبل، أنا على استعداد للعمل مع شخص لديه مشاكل في الصحة النفسية.
7. In the future, I would be willing to live nearby to someone with a mental health problem.	7. فيالمستقبل، أنا على استعداد للعيش بجوار شخص لديه مشاكل في الصحة النفسية.
8. In the future, I would be willing to continue a relationship with a friend who developed a mental health problem.	8. فيالمستقبل، أنا على استعداد لمواصلة علاقتي مع صديق ظهرت لديه مشاكل فيالصحةالنفسية.
